# Drug stockpiling behavior and its impact on anxiety among the general public in the early stage after the lifting of China’s Zero-COVID policy: results from a web-based survey

**DOI:** 10.3389/fphar.2025.1524068

**Published:** 2025-05-12

**Authors:** Yu Huang, Shuiyang Xu, Xiang Zhao, Lei Wang, Qiaohong Lv, Suxian Wu, Qingqing Wu, Xuehai Zhang

**Affiliations:** Department of Health Education, Zhejiang Provincial Center for Disease Control and Prevention, Hangzhou, China

**Keywords:** COVID-19, drug stockpiling, drug shortages, anxiety, drug management, China

## Abstract

**Background:**

On 7 December 2022, China lifted most of the restrictions under the so-called zero-COVID policy due to factors like less toxicity of the new variants of the virus, leading to widespread infections throughout China.

**Objectives:**

This study aims to assess the stockpiling behavior of COVID-19 medicines by the general population in Zhejiang at the early stage after China’s zero-COVID policy cancellation and its impact on people’s anxiety.

**Methods:**

A cross-sectional, internet-based survey was conducted to collect information on COVID-19 drug purchasing behavior, sociodemographic characteristics, anxiety levels, etc. Chi-square tests and univariate analyses were used to explore the association between COVID-19 medicines purchasing behavior and sociodemographic characteristics. Multivariate analyses were employed to explore the impact of COVID-19 drug purchasing behavior on anxiety status.

**Results:**

Among 38,480 participants, stockpiling behavior of COVID-19 medicines was reported by 35.74% of them and was most common among participants from Huzhou area, female, those who aged< 20 years, those with postgraduate education level, health workers. A total of 20,986 (54.54%) participants claimed that they were unable to access any COVID-19 medicines, while 3,742 (9.72%) participants felt it unnecessary to stockpile medicines. The majority of the participants (82.3%) experienced anxiety. Multivariate analyses found that compared to those with severe anxiety, those with moderate anxiety were 1.76 times more likely to have stockpiled COVID-19 medicine (aOR 1.76, 95% CI 1.64-1.89); those with mild anxiety were 2.11 times (aOR 2.11, 95% CI 1.98-2.24) more likely to have stockpiled COVID-19 medicine; those with no anxiety were 2.48 times (aOR 2.48, 95% CI 2.31-2.67) more likely to have stockpiled COVID-19 medicine.

**Conclusion:**

At the early stage after China’s zero-COVID policy cancellation, drug stockpiling among the public and the subsequent drug shortage was observed. There exists inequity in distribution between regions and among different groups of people. Many people experienced anxiety, especially those without access to COVID-19 medications. Measures for equitable drug distribution and public education on safe self-medication should be taken for future public health events.

## 1 Introduction

In December 2019, an outbreak of pneumonia with an unexplained origin was reported in Wuhan, Hubei Province of China (Lu et al., 2020; Hui et al., 2020). Soon, it was recognized as being triggered by a novel coronavirus (COVID-19). In only 30 days, COVID-19 spread at a fast pace from one city to the whole nation and then to other countries around the world (Wiersinga et al., 2020; Novel Coronavirus Pneumonia Emergency Response Epidemiology Team, 2020; Lim et al., 2020; Spina et al., 2020). The symptoms and manifestations of COVID-19 can be severe, with acute respiratory failure and sepsis, and sometimes even leading to a fatal outcome (Jernigan and CDC COVID-19 Response Team, 2020). On 30 January 2020, the World Health Organization (WHO) declared the outbreak a public health emergency of international concern (World Health Organization, 2020).

Initially, China adopted stringent measures to control COVID-19, namely the so-called dynamic zero-COVID policy. This resulted in long periods of national lockdowns, school closures, travel restrictions and the suspension of many crucial economic activities (The Lancet, 2021; Wei et al., 2021; Li and Zhang, 2023; Tang et al., 2023; Huang et al., 2023). On 7 December 2022, as the virulence of the new virus variants diminished and the prevalence of COVID-19 vaccines increased, the Chinese government determined to lift most of the restrictions (National Health Commission of the People’s Republic of China, 2022; Wilson and Flahault, 2023). This change in the COVID-19 policy was directly related to the accelerated spread of COVID-19, giving rise to widespread infections across China.

News and rumors spread via social media, television, and daily interactions regarding the seriousness of infections and the number of fatalities can give rise to anxiety and fear. Consequently, people act on their primal instincts and are prone to engage in panic buying to ease this psychological reactions (Arafat et al., 2020). Panic buying have had an impact on many items globally, such as groceries, food, sanitizers, disinfectants, and protective equipment, resulting in acute shortages of these items and leaving those in need unable to obtain them (Ma and Tsai, 2022; Brizi and Biraglia, 2021; Fabusoro et al., 2023; Micalizzi et al., 2021; Garbe et al., 2020). Above all, the loosened policy and the increased risk of infection may have influenced the people’s awareness of health protection, leading some individuals to stockpile drugs to ensure timely administration (Al Zoubi et al., 2021; Miah et al., 2024). A study conducted in Uganda, demonstrated that there was an increased purchase of antimicrobials by customers from community pharmacies between January 2018 and October 2021 (Kiragga et al., 2023). Increased drug purchases at the early stage of the COVID-19 pandemic were also recorded in developed countries like Germany and the United States (Callaway Kim et al., 2021; Kostev and Lauterbach, 2020). A time series analysis of country-level drug purchase data from 2014 to 2020 showed that before COVID-19, the standardized medication units per 100 population were 3,990–4,760 monthly. In March 2020, with rapid COVID-19 spread, there was a 15% increase in drug purchases to 5309.3 units per 100 population (Suda et al., 2003). The results indicate that COVID-19 already poses a significant challenge to the production and delivery of pharmaceutical products.

It should be noted that extensive panic buying of drugs increases the pressure on patients seeking medical care. This not only causes drug shortages but may also lead to psychological issues like death anxiety (Amerio et al., 2020; Ambrosetti et al., 2021). Panic buying of COVID-19 drugs may result in an increase in self-medication (SM) as a preventive measure. SM entails the use of medicines to treat self-recognized symptoms or diseases without consultation and the irrational use of over-the-counter drugs (Moussa et al., 2023). During the COVID-19 pandemic, the absence of a definite treatment led to increased SM (Stüdemann et al., 2024; Jirjees et al., 2022; Sadio et al., 2021). SM is a relatively widespread practice globally. In public health emergencies such as the COVID-19 epidemic, if properly implemented and under proper drug prescription laws, SM can empower patients to manage their health at home and improve their healthcare, thus alleviating the burden on healthcare facilities (Kiragga et al., 2023). However, previous research has found that SM is associated with increased rates of overdose, adverse drug reactions, and an increased risk of antimicrobial resistance (Author anonymous, 2018; Schmiedl et al., 2014; Alied, 2021).

Although numerous reports indicate that the COVID-19 pandemic have led to excessive and hasty purchases of various drugs, including antibiotics, to treat COVID-19 symptoms (Al Zoubi et al., 2021; Miah et al., 2024; Kiragga et al., 2023; Callaway Kim et al., 2021; Kostev and Lauterbach, 2020; Suda et al., 2003). There is still a lack of empirical evidence on COVID-19 drug purchases in China, especially during the early stages when the Chinese government relaxed its zero-COVID policy and people were most susceptible to anxiety and fear. The mental health survey in China in 2022 show that the prevalence rate of anxiety disorder among Chinese adults is 4.98%, and the prevalence rate of depressive disorder is 2.98%. This study aims to evaluate the behavior of the general population in Zhejiang, China regarding the stockpiling of COVID-19 medications in the early stage after the dynamic zero-COVID policy was canceled in China and its impact on people’s anxiety status.

## 2 Materials and methods

### 2.1 Data collection and quality control

The survey was conducted between December 16 and 30, 2022, among residents aged 18–69 years old in 11 administrative districts in Zhejiang, China. An online survey questionnaire was first sent to the 11 municipal centers for disease control and prevention. They promoted and carried out online investigations within their jurisdictions. The questionnaire was hosted on *wenjuan*. *Wenjuan* is a professional online questionnaire, test, assessment and voting platform, focusing on providing users with powerful and humanized online design questionnaires, data collection, customized reports, survey results analysis and other series of services.

The questionnaire was designed by the Zhejiang Provincial Center for Disease Control and Prevention. A pilot study was first conducted to check the reliability of the questionnaire. The questionnaire was then modified after feedbacks from the pilot study and two rounds of Delphi method collaboration, in which the face validity was checked by subject matter experts, and the literature was reviewed to assess for content validity. The questionnaire collected sociodemographic data, COVID-19 medicines purchased by participants, purchasing channels of COVID-19 medicine, participants’ main reasons for not purchasing COVID-19 medicines, anxiety levels, other COVID-19 related behaviour such as social restriction, frequent hand-washing, hoarding objects, et al.

To control the quality of the questionnaire, we set up a quality control question in the questionnaire to identify participants who are not taking the survey seriously. The data of these participants will not be included in the analysis.

### 2.2 Measurements control

#### 2.2.1 Anxiety

The Generalized Anxiety Disorder-7 (GAD-7) was used to for the assessment of anxiety. GAD-7 has confirmed validity and reliability. It consists of seven items, each rated on a 4-point scale: (0) not at all, (1) several days, (2) more than half the days, and (3) nearly every day. A higher total score indicates greater symptom severity. Scores ranging from 0 ∼ 4 are classified as no anxiety, 5 ∼ 9 as mild anxiety, 10 ∼ 14 as moderate anxiety, and 15 ∼ 21 as severe anxiety.

#### 2.2.2 Stockpiling behaviour of COVID-19 medicine

Individuals or households who had an act of purchasing the following medications beyond their immediate therapeutic needs at the early stage of the biggest wave of COVID-19 epidemic were considered to have a stockpiling behaviour of COVID-19 medicines in that period: (1) Fever reducers, (2) Pain relievers such as ibuprofen (Advil, Motrin IB, others) or acetaminophen (Tylenol, others), (3) Cough medicine, (4) Nasal congestion/snot relievers, (5) other medicines that may relieve the symptoms of COVID-19 infection.

### 2.3 Statistical analysis

Data were exported from the *wenjuan* website to Excel (Microsoft), and were analyzed using the Statistical Analysis System (SAS), version SAS Viya Long-Term Spport 2024.03 (SAS Institute Inc., Cary, NC, United States). Standard descriptive statistics were used for continuous and categorical variables to describe the characteristics of participants in this study. The Chi-square test was used to explore the association between COVID-19 medicines purchasing behavior and sociodemographic characteristics including age, gender, education level, and occupation. Univariate logistic regression analysis were conducted to identify the impact of sociodemographic characteristics on purchasing behaviour of COVID-19 medicine.

Multivariate logistic regression analysis were conducted to explore the association between purchasing behaviour of COVID-19 medicine and anxiety by controlling age, sex, education level, and occupation. The general form of the multivariate logistic regression model is:

$$\text{logit }\left(P\left(Y=1\right)\right)=\ln\left(1-P\left(Y=1\right)P\left(Y=1\right)\right)=\beta_{0}+\beta_{1}X_{1}+\beta_{2}X_{2}+\cdots +\beta_{p}X_{p}$$

*Y* is the binary dependent variable representing the purchasing behaviour of COVID-19 medicine, *X*1 represents the levels of anxiety; *X*2, *X*3,*X*4, *X*5 represents age, sex, education level, and occupation, respectively. Odds ratios with 95% confidence intervals were used to express measures of the associations. *P-*values less than 0.05 were considered to represent significance (two-sided).

### 2.4 Ethics approval

The Zhejiang Provincial Center for Disease Control and Prevention Ethics Board approved the study protocol. The survey was anonymous. Participants were informed on the purpose and characteristics of the study and provided informed consent by clicking a box.

## 3 Results

### 3.1 Respondents’ characteristics

A total of 40,130 residents participated in the survey. After quality control, 1,650 samples were excluded and 38,480 samples were included in the study. There were 10,431 males (27.11%) and 28,049 females (72.89%). 1,129 participants (2.93%) were aged 20 years old or below, 35,970 participants (93.48%) were aged 20-60, and 1,384 participants (3.60%) were over 60. There were 22,180 participants with education level of junior college/undergraduate, accounting for 57.64%, followed by those with education level of junior high school (18.79%), senior high school (18.24%), postgraduate or above (3.00%), primary school or below (2.34%). The proportion of health workers, government staff, students, farmers, Business or service industry/stay-at-home/unemployed were 7.92%, 20.04%, 3.96%, 3.76%, 64.32%, respectively; There were 31,053 married people (80.70%) and 7,427 unmarried people (19.30%). A total of 2,873 people (7.47%) resided in households with confirmed COVID-19 cases. The detailed demographic characteristics of parents are presented in Table 1.

**TABLE 1:** Sociodemographic characteristics of participants completing the online survey (N = 38,480).

Characteristic	N	%
Gender		
Male	10,431	27.11
Female	28,049	72.89
Age (years)		
<20	1,126	2.93
20-60	35,970	93.48
>60	1,384	3.60
Education level		
Primary school	900	2.34
Junior high school	7,229	18.79
Senior high school	7,017	18.24
Junior college/undergraduate	22,180	57.64
Postgraduate	1,154	3.00
Occupation		
Health workers	3,049	7.92
Government staff	7,712	20.04
Students	1,522	3.96
Farmers	1,447	3.76
Business or service industry/stay-at-home/unemployed, etc.)	24,750	64.32
Marital status		
Married	31,053	80.70
Others (single/divorced/widowed/separated, etc.)	7,427	19.30
Family members infected with COVID-19		
Yes	2,873	7.47
No	35,607	92.53

### 3.2 Stockpiling behavior of COVID-19 medicines in Zhejiang at the early stage after China’s zero-COVID policy cancellation

**FIGURE 1:**
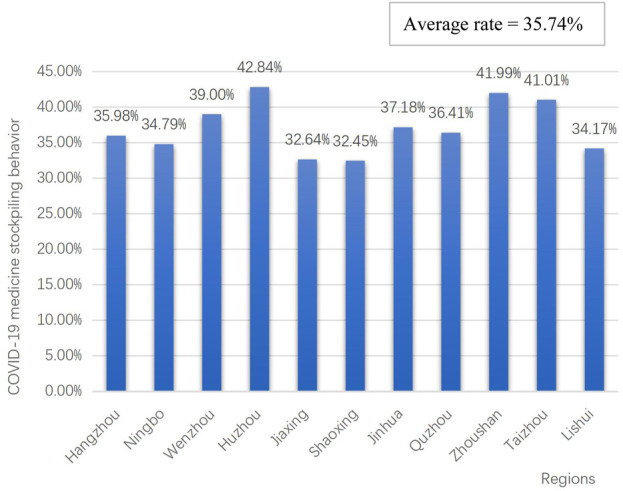
COVID-19 medicine stockpiling behavior in Zhejiang at the early stage after zero-COVID policy cancellation in China.

Figure 1 shows that 35.74% (13,752/38,480) of the participants from 11 administrative units in Zhejiang reported stockpiling behavior for COVID-19 medicines in the early stage after the cancellation of China’s zero-COVID policy. Participants from Huzhou area reported the highest rate of drug stockpiling at 42.84% (634/1,480), followed by Zhoushan at 41.99% (1,035/2,465), Taizhou at 41.01% (760/1853), Wenzhou at 39.00% (814/2087), Jinhua at 37.18% (383/1,030), Quzhou at 36.41% (611/1,678), Hangzhou at 35.98% (3,096/8,604), Ningbo at 34.79% (1,371/3,941), Lishui at 34.17% (654/1914), Jiaxing at 32.64% (3,708/11,361), and Shaoxing at 32.45% (368/1,134). The Chi-square test for comparing multiple sample rates found that generally speaking, there are differences among the population rates (P <.0001).

**FIGURE 2:**
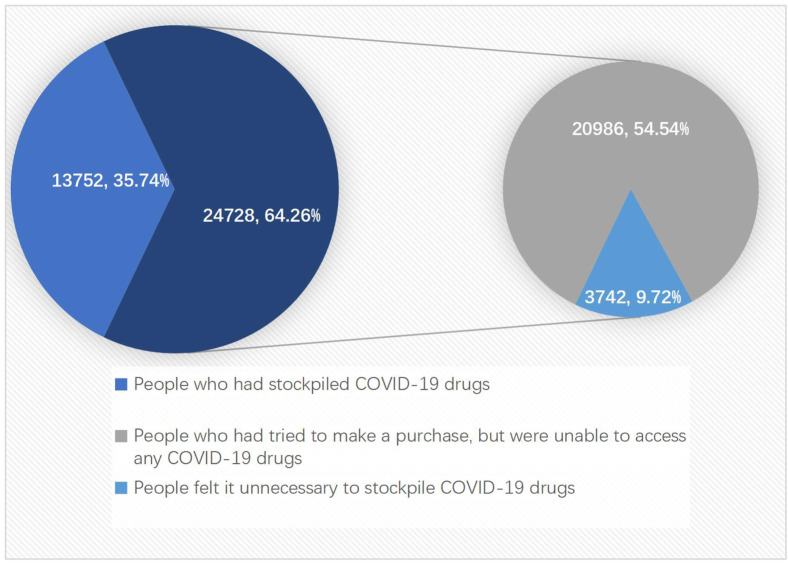
Proportions of participants who had stockpiled COVID-19 drugs and reasons for those not purchasing them.

Figure 2 shows that 24,728 participants had not stockpiled COVID-19 drugs, making up 64.26% of the total participants (24,728/38,480). Regarding the reasons for not stockpiling drugs, 20,986 participants (54.54%) claimed that they had attempted to make a purchase but were unable to obtain any of the drugs. Additionally, 3,742 participants (9.72%) felt it was unnecessary to stockpile COVID-19 drugs.

**FIGURE 3:**
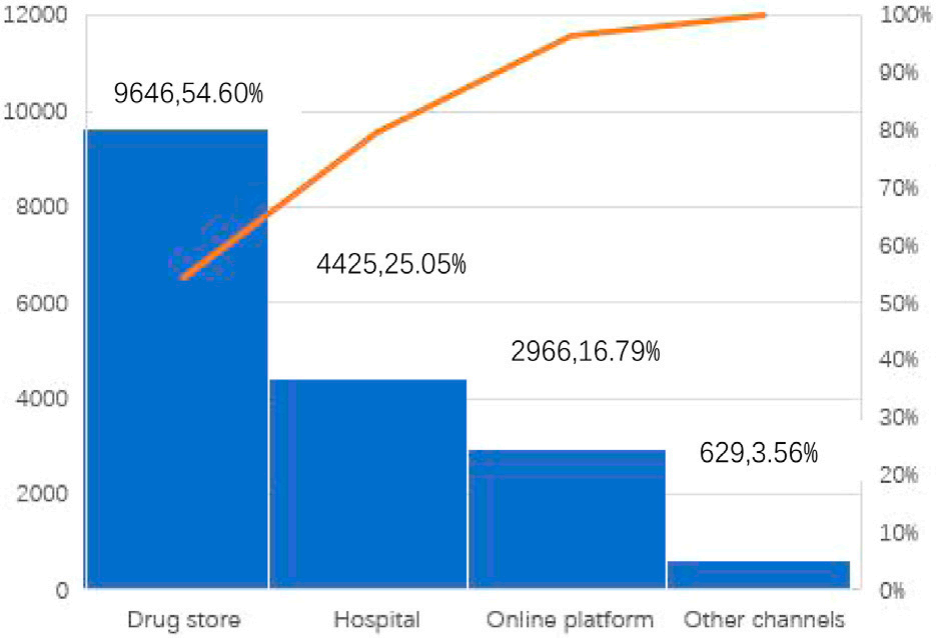
Channels of COVID-19 medicine purchasing among participants.

As can be seen from Figure 3, drug stores are the main channel for people to buy COVID-19 drugs in the early stage after the cancellation of China’s zero-COVID policy. A total of 9,646 participants purchased medicines through pharmacies, accounting for 54.60% of all purchasing channels. Hospitals are the second most important means for people to purchase COVID-19 medicines, accounting for 25.05%. The Internet has also become a way for people to buy medicines, accounting for 16.79%. Other ways of obtaining COVID-19 medicines accounted for 3.56%.

### 3.3 Behaviour of COVID-19 drug stockpiling by gender, age, education level and occupation

As shown in Table 2, there were statistically significant differences in the stockpiling behavior of COVID-19 medicine among participants by sex, age, education level, and occupation (P <.0001). The stockpiling rate was 38.58% for women and 28.09% for men (OR 1.61, 95% CI 1.53–1.69). The stockpiling rates of participants under 20 years old, between 20 and 60 years old, and over 60 years old were 52.49%, 35.49%, and 28.54% respectively. Compared with people over 60 years of age, those aged under 20 years (OR 2.77, 95% CI 2.34–3.26) and between 20 and 60 years old (OR 1.38, 95% CI 1.22–1.55) were more likely to stockpile COVID-19 medicine. The stockpiling rates of COVID-19 medicine among participants with education levels of primary school, junior high school, senior high school, junior college/undergraduate, and master’s degree groups were 24.78%, 28.05%, 29.94%, 39.87%, and 48.27% respectively. As the education level increased, the chance of stockpiling COVID-19 medicine among participants also increased. Compared to people with primary school education, those with junior high school education (OR 1.18, 95% CI 1.01–1.39), senior high school education (OR 1.30, 95% CI 1.11–1.52), junior college/undergraduate education (OR 2.01, 95% CI 1.73–2.35), and postgraduate education (OR 2.83, 95% CI 2.34–3.42) had an increasingly higher opportunity to stockpile COVID-19 medicine. Compared to farmers, health workers (OR 4.12, 95% CI 3.58–4.74), government staff (OR 2.48, 95% CI 2.18–2.82), and students (OR 2.84, 95% CI 2.43–3.33) had a higher possibility of having COVID-19 medicine.

**TABLE 2:** Behaviour of COVID-19 medicine purchases by gender, age, education level and occupation.

Variables	COVID-19 drug stockpiling behavior		COVID-19 drug stockpiling rate (%)	χ^2^	*P*	OR (95%CI)
	Yes	No				
Sex						
Male	2,930	7,501	28.09	364.53	<0.0001	1
Female	10,822	17,227	38.58			1.61 (1.53,1.69)
Age (years)						
<20	591	535	52.49	169.71	<0.0001	2.77 (2.34,3.26)
20-60	12,766	23,204	35.49			1.38 (1.22,1.55)
>60	395	989	28.54			1
Education level						
Primary school	223	677	24.78	579.31	<0.0001	1
Junior high school	2028	5,201	28.05			1.18 (1.01,1.39)
Senior high school	2,101	4,916	29.94			1.30 (1.11,1.52)
Junior college/undergraduate	8,843	13,337	39.87			2.01 (1.73,2.35)
Postgraduate	557	597	48.27			2.83 (2.34,3.42)
Occupation						
Health workers	1724	1,325	56.54	1,260.61	<0.0001	4.12 (3.58,4.74)
Government staff	3,388	4,324	43.93			2.48 (2.18,2.82)
Students	720	802	47.31			2.84 (2.43,3.33)
Business or service industry/stay-at-home/unemployed, etc.)	7,573	17,177	31.52			1.40 (1.23,1.58)
Famers	347	1,100	23.98			1

### 3.4 Association between anxiety and behaviour of COVID-19 medicine purchase

Using the GAD-7 scale to measure the anxiety level of the participants, it was found that 31,668 (82.30%) out of 38,480 participants had anxiety. Among them, 16,295 (42.34%) had mild anxiety, 7,274 (18.90%) had moderate anxiety, and 8,099 (21.05%) had severe anxiety. The proportions of COVID-19 medicine stockpiling behavior among participants with no anxiety, mild anxiety, moderate anxiety, and severe anxiety were 43.12% (2,937/6,812), 38.92% (6,342/16,295), 35.61% (2,590/7,274), and 23.25% (1,883/8,099) respectively.

**FIGURE 4:**
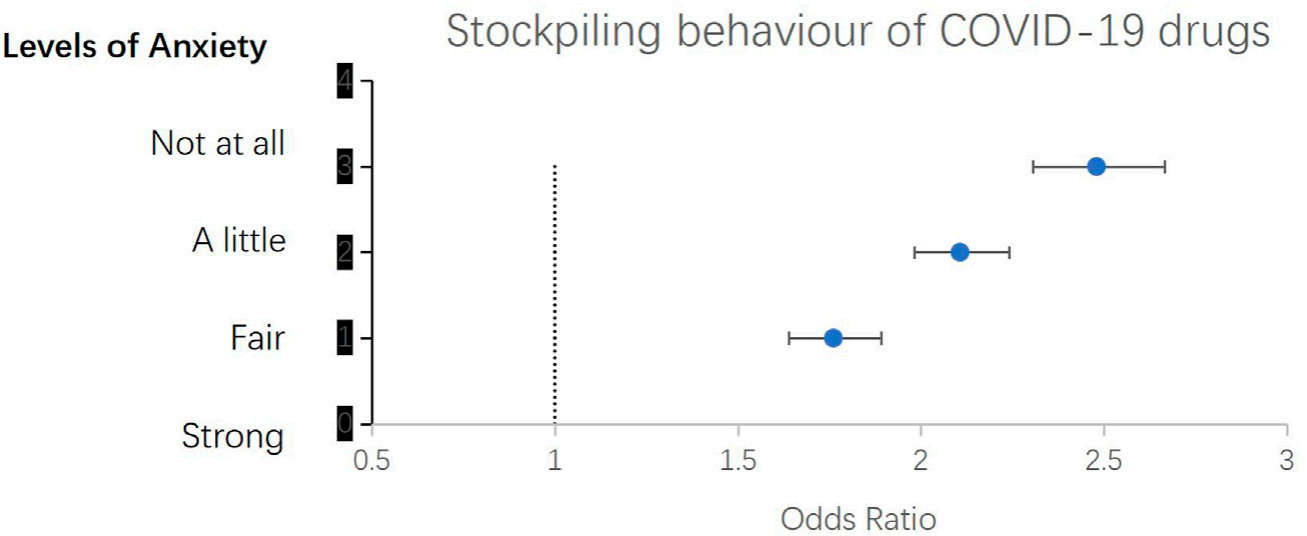
The association between the stockpiling behavior of COVID-19 drugs and the anxiety status of participants in the early stage after the cancellation of China’s zero-COVID policy.

As shown in Figure 4, by using multivariate Logistic regression analysis and after controlling for gender, age, education level, and occupation, it was discovered that there was a statistically significant correlation between COVID-19 medicine stockpiling and the anxiety level of the participants. Participants who had purchased COVID-19 drugs were less likely to be anxious. Compared to those with severe anxiety, those with moderate anxiety were 1.76 times more likely to have stockpiled COVID-19 medicine (aOR 1.76, 95% CI 1.64–1.89); those with mild anxiety were 2.11 times (aOR 2.11, 95% CI 1.98–2.24) more likely to have stockpiled COVID-19 medicine; and those with no anxiety were 2.48 times (aOR 2.48, 95% CI 2.31–2.67) more likely to have stockpiled COVID-19 medicine.

## 4 Discussion

### 4.1 Principle findings

This study was undertaken to evaluate the behavior of the general population in Zhejiang, China with respect to the stockpiling of COVID-19 medicines in the early stage following the cancellation of the zero-COVID policy in China, as well as its impact on people’s anxiety status. The major strength of our study lies in its originality. Given the dearth of similar studies, this research will serve as a foundation for future studies that highlight such crucial issues in the context of future public health emergency.

The results of our study revealed that 35.74% of the participants had stockpiled COVID-19 medicines. This behavior can be explained as a coping mechanism during stressful circumstances, aiming to maintain the health of one’s family or to act in a manner similar to other community members (Kostev and Lauterbach, 2020; Yuen et al., 2020; Chua et al., 2021). Psychologically, it may be driven by a fear of drug shortages, price increases, the surge in infections, and the availability of medicines and home delivery services during the pandemic (Miah et al., 2024; Jifar et al., 2022). A study from Jordan reported a higher prevalence of drug stockpiling (44.3%) during the COVID-19 pandemic (Al Zoubi et al., 2021). Stockpiling occurs not only among individuals and families but also at the national level in emergencies. For instance, in the early stage of the COVID-19 pandemic, the United States decided to stockpile remdesivir by purchasing over 500,000 doses (Dawoud et al., 2020).

The findings of our study demonstrated that female gender, younger age, higher education level, and being a health worker were associated with a higher prevalence of stockpiling COVID-19 medicines. This indicates that socioeconomic status influences the patterns of panic buying of COVID-19 drugs. In many studies, women have been confirmed to have greater concern for their own and their family’s health, which may lead to a higher prevalence of drug stockpiling among them (Huang et al., 2020; Huang et al., 2023). The online platform is a crucial channel for accessing COVID-19 drugs, especially when pharmacies are experiencing shortages. Young people are proficient in using the Internet and have an advantage over older people in purchasing medicines online (Thayer and Ray, 2006; Sözeri Öztürk et al., 2023; Estacio et al., 2019). This could be the main reason why younger people in this study have more drug purchases than older ones. The correlation between people with a higher education level and a higher prevalence of stockpiling COVID-19 medicines may be because they typically have more socioeconomic resources that assist them in accessing pharmaceutical products (Elek et al., 2021). In a study conducted in Brazil, higher socioeconomic status and younger age were also found to be linked to a higher prevalence of drug stockpiling. However, on the contrary, in that study, male gender was found to have a higher prevalence of drug purchasing than female (Lins and Aquino, 2020). This gender difference may be attributed to the different roles that Chinese women play in the family. Finally, the study revealed that health workers have the highest drug purchase rate among all occupations. The professional nature of medical personnel leads to greater access to medicines, which indicates that measures for equitable distribution of medicines through a resilient drug supply chain should be implemented to enhance the national response to future unforeseen events.

China, like the United Kingdom, New Zealand, the European Union, Australia, the United States and some other countries, all adopt the centralized drug procurement model. Usually, the public sector play a leading role in the process of centralized drug procurement in countries like New Zealand, the European Union and Australia. However, the United States mainly entrust the private sector Group Purchasing Organizations to conduct price negotiations and centralized procurement. In China, the state organizes centralized procurement of drugs through volume - based procurement (Yang, 2020). In 2021, in light of the experience of responding to challenges posed by the COVID-19 epidemic, China revised the Measures for the Management of National Pharmaceutical Reserves. This revision clarified the tasks, plan management, procurement, reserve, utilization, and fund management of reserve units, and expanded the conditions and forms of reserve enterprises. As a result, a theoretically complete national medicine reserve management system has been established (Ministry of Industry and Information Technology of the People’s Republic of China, 2021). The “14th Five-Year Plan for the Development of Pharmaceutical Industry” released in 2022 also made plans for strengthening the construction of the national pharmaceutical reserve system (The Central People’s Government of the People’s Republic of China, 2021). In this study, through a large-scale online survey, it was found that over half of the respondents were unable to access any COVID-19 medicines. This suggests that although China has made sufficient preparations, a sudden surge in demand still resulted in a shortage of drugs. Currently, there are no research reports on the same topic in Zhejiang Province or even across the country. Optimal treatment of viral infections requires timely administration of drugs, and drug delivery and proper storage of drugs also serve as a crucial means to ensure such timely administration (Koonin and Patel, 2018). When access to medicines is hindered, drug production and delivery becomes essential for improving access to medicines and achieving better treatment outcomes during epidemics (Klarman et al., 2023). The results of this study provide scientific evidence for optimizing and improving relevant drug security measures when dealing with similar public health events in China in the future.

The COVID-19 pandemic has impacted human life in many ways, one of which is the psychological factor (Metin et al., 2022; Saeed et al., 2022; Delpino et al., 2022). The uncertainties during the pandemic have caused anxiety. A meta-analysis on this matter revealed that healthcare workers had the highest prevalence of anxiety (36%), followed by university students (34.7%), the general population (34%), teachers (27.2%), parents (23.3%), pregnant women (19.5%), and police (8.79%) (Saeed et al., 2022). Our study found a much higher prevalence of anxiety (82.30%). Such a high prevalence of anxiety may be related to the specific period when the survey was conducted. At that time, the Chinese government canceled the zero-COVID policy that had lasted for 3 years, leading to the large-scale spread of COVID-19. Many factors might have influenced the anxiety status. The study found an association between the stockpiling of COVID-19 drugs and the anxiety levels among participants. Multivariate analysis indicated that as the drug stockpiling rate increased, the anxiety level among the participants decreased. Compared to those with severe anxiety, individuals with moderate anxiety were 1.76 times more likely to have purchased COVID-19 medicine (aOR 1.76, 95% CI 1.64-1.89); those with mild anxiety were 2.11 times more likely (aOR 2.11, 95% CI 1.98-2.24); and those with no anxiety were 2.48 times more likely (aOR 2.48, 95% CI 2.31-2.67) to have purchased COVID-19 medicine. These findings suggest that the purchasing behavior of drugs is an anxiety reaction, and stockpiling drugs can to some extent alleviate anxiety. A risk communication strategy that enables real-time exchange of information, advice, and opinions is needed to assist people in making informed decisions to mitigate their fear and anxiety in future emergency preparedness and response, thereby reducing panic buying.

Finally, it is noteworthy that with the progression of the COVID-19 epidemic, an increasing number of infected people may turn to SM for symptomatic treatment. Along with the increasing SM behavior, the inherent risks associated with this act, such as drug-drug interactions, adverse events, drug toxicity, and masking of symptoms, may have also increased. Studies have demonstrated that adverse reactions of SM frequently occur. For instance, the rate of adverse reactions caused by elderly people taking hypertension drugs on their own is nearly 10% (Chen et al., 2019). SM to treat COVID-19 symptoms has led to drug misuse, which in some cases has resulted in the development of fatal adverse drug reactions. Moreover, the stockpiling and misuse of antibiotics by a large number of people increase the risk of antimicrobial resistance and may give rise to new public health problems (Yang and Yi, 2020). The prevalence of SM with antibiotics during COVID-19 in the Eastern Mediterranean Region Countries ranged from 20.8% to 45.8% among different studies (Jirjees et al., 2022). It is urgently necessary for the CDC, hospitals, mass media, pharmacies, and others to collaborate in educating the general public about the hazards of excessive drug usage and safe SM practices, especially the proper use of antibiotics during the COVID-19 pandemic and future public health events. Additionally, there is a need for stringent restrictions on dispensing antibiotics from community pharmacies and hospitals during such events.

### 4.2 Limitations

This study has certain limitations. Firstly, to quickly obtain the drug stockpiling behavior of the general population, we kept the questionnaire and investigation period short, which limited the depth of the study. Secondly, among the participants in the online survey, the participation of women and those with a higher education level is significantly higher than that of men and those with a low education level. Directly extending the survey results to the entire population may cause bias. Thirdly, cross-sectional studies can only explore associations between drug stockpiling and anxiety levels, rather than establish cause and effect relationships.

## 5 Conclusion

We observed evidence of drug stockpiling in the early stage after the cancellation of zero-COVID policy in Zhejiang, China, especially among health workers, young people, and those with a higher education level. Pharmacies, hospitals, and online platforms are the main channels for purchasing COVID-19 drugs. However, over half of the participants still could not obtain any COVID-19 drugs. The vast majority of participants experienced anxiety, especially those who were unable to access COVID-19 drugs. Obtaining COVID-19 drugs has a positive impact on people’s anxiety levels. Measures for the equitable distribution of medicines through a resilient drug supply chain should be taken to enhance the national response to future public health events. Furthermore, the behavior of purchasing COVID-19 medicines may lead to an increase in SM as a preventive measure. Educating the general public about the hazards of excessive drug usage, safe self-medication practices, especially the proper use of antibiotics, should be considered to respond to future public health emergencies like COVID-19.

## Data Availability

The raw data supporting the conclusions of this article will be made available by the authors, without undue reservation.

## Abbreviations

SM, Self-Medication; GAD-7, Generalized Anxiety Disorder-7; OR, odds ratio; AOR, adjusted odds ratio; 95%CI, 95% confidence interval.
